# Effect of miR-34a in regulating steatosis by targeting PPARα expression in nonalcoholic fatty liver disease

**DOI:** 10.1038/srep13729

**Published:** 2015-09-02

**Authors:** Jiexia Ding, Meng Li, Xingyong Wan, Xi Jin, Shaohua Chen, Chaohui Yu, Youming Li

**Affiliations:** 1 Department of Infectious Diseases, Hangzhou First People’s Hospital, No. 261 Huansha Road, Hangzhou 310006, Zhejiang Province, China; 2 Department of Gastroenterology, The First Affiliated Hospital, Medical School, Zhejiang University, No. 79 Qingchun Road, Hangzhou 310003, Zhejiang Province, China

## Abstract

MicroRNA-34a (miR-34a) is thought to be involved in nonalcoholic fatty liver disease (NAFLD). However, the association between altered expression of miR-34a and the pathophysiological features of NAFLD remains unclear. Here, we investigated the mechanisms by which miR-34a influences NAFLD through the PPARα-related pathway. Real-time quantitative PCR, western blotting and other assays kit were used to investigate the expression and function of miR-34a in an NAFLD model. Cultured cells transfected with miR-34a inhibitor and C57BL/6 mice injected with the miR-34a inhibitor through vein tail were conducted for the effects of miR-34a on its target. MiR-34a levels were significantly upregulated in steatosis-induced hepatocytes and in liver tissues of high-fat diet-fed mice. The upregulation of miR-34a resulted in the downregulation of hepatic PPARα and SIRT1 that are the direct targets of miR-34a. Silencing miR-34a led to an initially increased expression of PPARα, SIRT1 and PPARα’s downstream genes. Activation of the central metabolic sensor AMPK was also increased. The miR-34a inhibitor suppressed lipid accumulation and improved the degree of steatosis. Taken together, our data indicated that decreased expression of miR-34a potentially contributes to altered lipid metabolism in NAFLD. Downregulation of miR-34a may be a therapeutic strategy against NAFLD by regulating its target PPARα and SIRT1.

Nonalcoholic fatty liver disease (NAFLD), one of the leading causes of chronic liver disease worldwide, comprises a spectrum of diseases, ranging from simple steatosis to steatohepatitis (NASH). Patients with NASH may develop cirrhosis and hepatocellular carcinoma (HCC). However, the pathogenesis of NAFLD remains largely unknown^1^. Recently, a new class of RNA regulatory genes, known as microRNAs (miRNAs), has introduced a new layer of gene regulation in eukaryotes. Mature miRNAs are a class of naturally occurring, small non-coding RNA molecules, about 21–25 nucleotides in length. They function to downregulate gene expression by translational repression, mRNA cleavage and deadenylation^2^,^3^. They are receiving growing attention because of numerous reports of their dysregulation in human diseases, and their potential as diagnostic and therapeutic targets. Several miRNAs have been described as the important regulators of liver pathophysiology, including NAFLD, cirrhosis and HCC^4^,^5^,^6^.

MiRNAs also have crucial functions in metabolic regulation and are aberrantly expressed in metabolic disease^7^,^8^. Recently, miR-34a has been reported to emerge as a specific miRNA modulated in liver disease. Hepatic miR-34a levels are highly elevated in both dietary and leptin-deficient ob/ob obese mice^9^. Consistent with these initial findings, miR-34a is increased in patients of NAFLD^10^,^11^ as well as in a mouse model of steatohepatitis^11^,^12^,^13^. Furthermore, miR-34a suppresses SIRT1, which regulates the activity of AMP kinase (AMPK), a known regulator of energy metabolism^14^.

The peroxisome proliferator-activated receptor-α (PPARα) is a member of the nuclear receptor superfamily. Upon ligand binding, PPAR forms a heterodimer with the retinoid X receptor, interacts with PPAR response elements in the target genes, and regulate their expressions^15^,^16^,^17^. Of three PPAR isoforms, PPARα is essential to modulate lipid transport and metabolism, mainly through activating mitochondrial and peroxisomal fatty acid β–oxidation pathways. PPARα regulates the constitutive transcription of genes encoding fatty acid (FA)-metabolizing enzymes and mitochondrial FA oxidation (FAO) activity, primarily in the liver^18^. PPARα activators, such as the widely prescribed fibrate drugs, ameliorate hepatic steatosis through enhancing mitochondrial FAO in mice^19^. Furthermore, PPARα exhibits an anti-inflammatory effect after feeding with a high-fat diet (HFD)^20^.

Here, we report that elevated miR-34a in NAFLD directly targets the transcription factor PPARα. Furthermore, *in vitro* and *in vivo* silencing of miR-34a in free fatty acid (FFA) or HFD-induced NAFLD models demonstrated the therapeutic feasibility of targeting miR-34a to treat NAFLD.

## Materials and Methods

### Cell lines, cell culture, *in vitro* model of cellular steatosis and miRNA transfection

The human normal hepatocyte cell line L02 cells was obtained from the Shanghai Institute of Cell Biology, Shanghai, China, and were cultured in Dulbecco’s modified eagle medium (DMEM) cell culture media supplemented with 10% (v/v) Fetal Bovine Serum(FBS) (Gibco, California, USA) under an atmosphere of 5% CO2 at 37 °C. Fat overloading induction of cells was done mainly according to the method in which L02 cells was exposed to a mixture of FFA (oleate and palmitate) at a final ratio of 2:1 and final concentration of 1 mM.

### miRNA and small interfering RNA transfection

To assess the influence of miR-34a inhibitor on cellular steatosis, cells were treated with FFA after 24 hours of miR-34a inhibitor transfection and harvested after 24 hours incubation with FFA. The day before transfection, the cells were plated in growth medium without antibiotics at a density of 30–40%. The transfection of hsa-miR-34a inhibitor (5′- ACA ACC AGC UAA GAC ACU GCC A-3′), chemically synthesized by Invitrogen (Carlsbad, USA) was performed using Lipofectamine 2000 (Invitrogen, Carlsbad, USA) according to the manufacturer’s protocol. The nonsense single-strand RNA (5′-CAG UAC UUU UGU GUA GUA CAA-3′), chemically synthesized by Invitrogen (Carlsbad, USA) were transfected into the negative control (NC) group. The 5′ end of the fluorescence-labeled miR-34a inhibitor was used to determine the transfection efficacy. The cell images were obtained using a phase-contrast microscope at an appropriate magnification (Olympus, Japan).

### Animals models of NAFLD

Male C57BL/6 mice, 4 weeks of age, each weighing 18–20 g, were purchased from Vital River and acclimated for 1 week after arrival before they were used for experiments. All mice were bred in-house in a pathogen-free facility and maintained in a 12-hour day/night cycle at controlled room temperature and provided free access to food and water. After one week of acclimatization to laboratory conditions, the mice were then randomly divided into two groups: (1) control group (n = 18): animals treated with the standard chow diet (SCD, 8% rice bran, 51% maize, 30% soybean powder, 3% bone powder, 1.3% multivitamin, and 6.7% mineral); (2) HFD group (n = 18): animals given a HFD (75% SCD, 2% cholesterol, 15% lard and 8% yolk powder). Six mice of each group were sacrificed at weeks 4, 8, and 12 after being placed on the respective diet. Livers were quickly excised, cleaned completely with ice-cold phosphate-buffered saline (PBS), and preserved in liquid nitrogen until use. Pieces of the remaining liver tissues were processed for histology. All animal studies were approved by the Animal Care and Use Committee of Zhejiang University in accordance with the Chinese guidelines for the care and use of laboratory animals.

### Tail vein injection of lentiviral vector

Male C57BL/6 mice, 4 weeks of age, each weighing 18–20 g, were purchased from Vital River and acclimated for 1 week after arrival before they were used for experiments. The sequence of miR-34a inhibitor was built to pLenti6.3/TO/V5 vector (Invitrogen, Carlsbad, USA), the virus titer was 2 × 10^8^ TU/ml. The mice had the standard chow (control group, SCD) or a HFD for 4 weeks. After 4 weeks, the HFD group was divided into three groups: one was kept in the same conditions, a third group was maintained on HFD injected with 100 μl of pLenti6.3/TO/V5 vector via the tail vein and a forth group was maintained on HFD injected with an equal volume of negative control siRNA (NC) for 4 more weeks. The animals were then sacrificed. Blood was collected just before sacrifice for serum biochemical analysis. Livers were quickly excised, cleaned completely with ice-cold PBS, and preserved in liquid nitrogen until use. Pieces of the remaining liver tissues were processed for histology and Oil red O staining.

### Oil red O staining, triglyceride level assay and H&E staining

Intracellular neutral lipids were stained with Oil red O and mice liver pathological damage was measured by hematoxylin and eosin (H&E) staining, as described in the Supplementary materials and methods. Intracellular and liver triglycerides were determined using a triglyceride assay kit (GPO-POD; Applygen Technologies Inc., Beijing, China), according to the manufacturer’s instructions.

### Real-time quantitative PCR (qPCR) and western blotting analyses

Procedures of RNA extraction, qPCR and western blotting were described in the Supplementary data.

### MiR-34a target prediction and validation

MiR-34a target prediction was carried out using the algorithms Target Scan Human V6.2 and PicTar. To validate the candidate miR-34a predicted targets, luciferase reporter assays were carried out. More detailed protocols are described in the Supplementary data.

### Statistical analysis

Results are expressed as means ± standard deviation. The significance of the difference in means was determined by a two-tailed Student *t* test and ANOVA test. Values of *P* < 0.05 were considered significant and are indicated by asterisks in the figures.

## Results

### Increased levels of miR-34a in L02 cells with FFA and C57BL/6 mice with HFD

**Figure 1:**
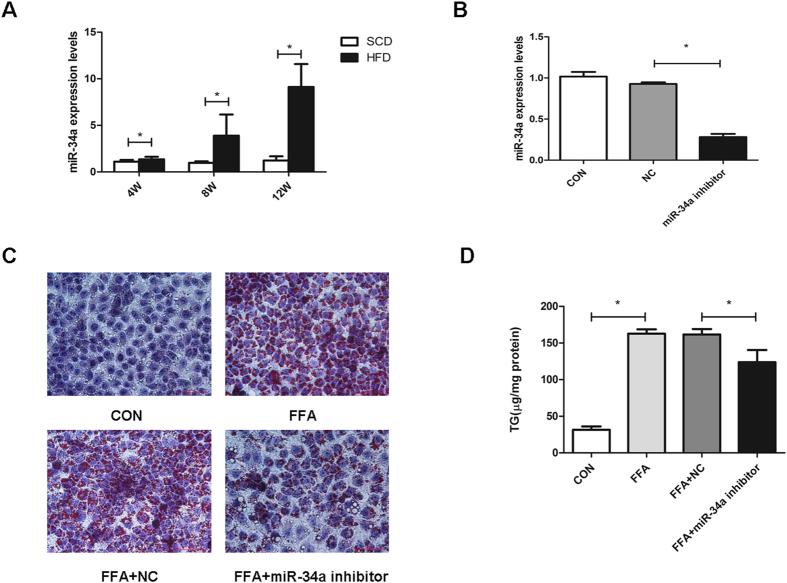
The expression of miR-34a in C57BJ/6 mice of NAFLD models and lipid accumulation improvement following inhibition of miR-34a in L02 cells. (**A**) The expression of miR-34a in C57BJ/6 mice fed with HFD or SCD. (**B**) The miR-34a inhibitor markedly reduced the miR-34a expression levels in L02 cells. Determined by qPCR, the data were normalized against U6 expression. (**C**) Oil red O staining showed the increase of lipid droplets caused by FFA was markedly attenuated in cells transfected with the miR-34a inhibitor (400×). (**D**) Influence of the miR-34a inhibitor on intracellular triglyceride level induced by FFA overload. Representative results from three independent experiments are shown. Data are mean ± standard deviation in A, B and D. *P < 0.05.

Q-PCR demonstrated significantly higher expression of miR-34a in FFA-induced L02 cells compared with the controls (Figure S1A). Intriguingly, there was a correlated increase of miR-34a expression in mice when the HFD feed time was extended from 4 weeks to 12 weeks (Fig. 1A).

### Inhibition of miR-34a expression alleviates hepatocellular steatosis *in vitro*

L02 cells transfected with 50 nM miR-34a inhibitor or NC were cultured in full media with or without FFA mixtures. There was an approximately 67% reduction of the miR-34a level in L02 cells treated with the inhibitor (Fig. 1B). As depicted in Fig. 1C, Oil red O staining demonstrated that increased lipid stores were present in hepatocytes treated with FFA for 24 hours. The increases were markedly attenuated in cells transfected with the miR-34a inhibitor. As expected, L02 cells transfected with miR-34a inhibitor resulted in a significant (23%) reduction in cellular triglyceride compared with NC after FFA exposure *(P* < 0.05) (Fig. 1D). Collectively, these results demonstrated that miR-34a could regulate the steatosis level in FFA-cultured L02 cells.

### Inhibition of miR-34a expression alleviates hepatic steatosis *in vivo*

**Figure 2:**
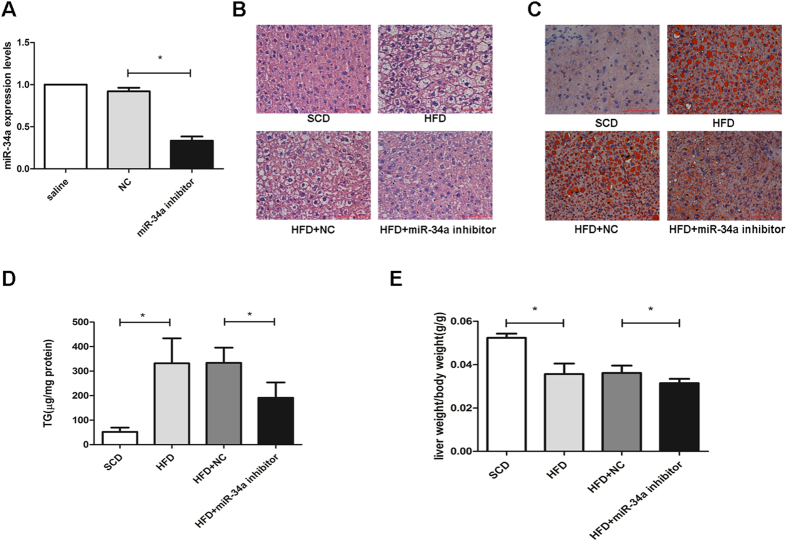
Inhibition of miR-34a attenuated hepatic steatosis and plasma AST levels in HFD-fed mice. (**A**) The miR-34a inhibitor markedly reduced the miR-34a expression levels in mice. (**B**) H&E-stained liver sections from mice fed with HFD treated with NC or miR-34a inhibitor (400×). (**C**) Oil red O staining of liver tissues fed with HFD, HFD treated with NC or miR-34a inhibitor respectively (400×). (**D**) Influence of miR-34a inhibitor on liver TG levels induced by HFD. (**E**) Influence of miR-34a inhibitor on liver weight/body weight ratio (liver index) induced by HFD. Representative results from three independent experiments are shown. Data are mean ± standard deviation in A, D and E. *P < 0.05.

To further evaluate the effect of miR-34a on HFD-induced hepatic steatosis *in vivo*, we adopted the ‘lentiviral tail vein injection’ method to deliver the miR-34a inhibitor into mouse hepatocytes. C57BL/6 mice that had been fed with a high-fat diet for 4 weeks were treated with 100 μl saline, miR-34a inhibitor at 2*10^7^ TU of pLenti6.3/TO/V5 vector or NC. There was an approximately 63% reduction in the miR-34a level in mouse liver tissues (Fig. 2A). As shown in Fig. 2B H&E staining and Fig. 2C Oil Red O staining demonstrated that the miR-34a inhibitor attenuated the steatosis degree in the liver tissues. In accordance with morphological findings, hepatic TG was reduced by 35% in mice injected with the miR-34a inhibitor, compared with the NC after 4 weeks of an HFD diet (*P* < 0.05) (Fig. 2D). Furthermore, liver weight/body weight ratio (liver index) reduced significantly in the miR-34a inhibitor groups (Fig. 2E). Liver inflammation or fibrosis was not significant observed in these experimental groups.

Serum levels of aspartate aminotransferase (AST) and alanine aminotransferase (ALT) have been regarded as markers of liver injury. The HFD resulted in a significant (*P* < 0.01) increase in the circulating levels of AST and total cholesterol (TC). However, HFD had no significant effects on ALT and TG. The levels of AST were significantly reduced in HFD-induced hepatic steatosis mice treated with miR-34a inhibitor compared to HFD-induced hepatic steatosis mice treated with NC duplex, indicating that the miR-34a inhibitor could attenuate liver injury (Figure S1B and Figure S1C).

### The lipid pathway transcriptional regulator PPARα is a direct target of miR-34a in the liver

**Figure 3:**
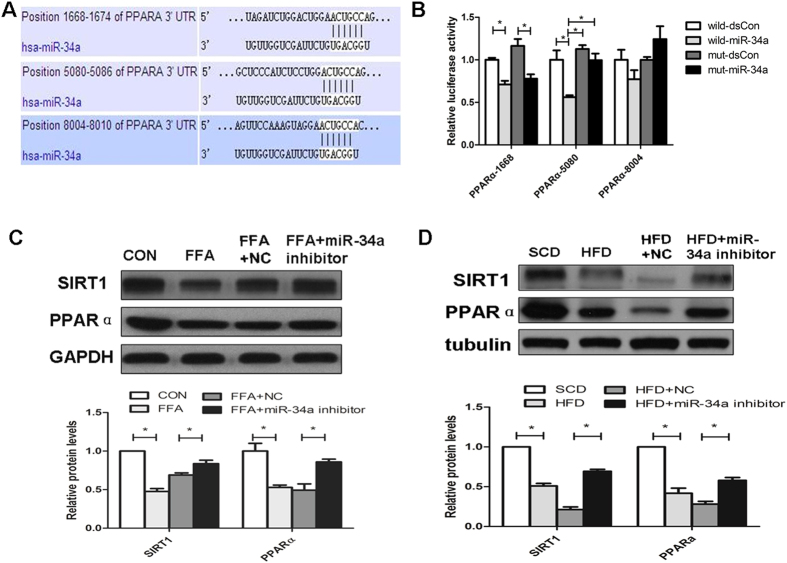
MiR-34a Target genes prediction, verification and the regulation of miR-34a on PPARα in L02 cells. (**A**) Seed sequence base-pairing between miR-34a and 3′ UTRs of PPARα as predicted by TargetScan. (**B**) The three regions of the 3′ UTR of PPARα that were subcloned into the luciferase reporter and mutations of the seed sequences. (**C,D**) Decreased protein expression of PPARα and SIRT1 were observed in steatosis L02 cells and HFD C57BL/6 liver tissues, and increased in the miR-34a inhibitor treated group in L02 cells and liver tissues. The PPARα and SIRT1 protein expression were detected by western blotting. GAPDH and tubulin were detected as a loading control. The results were standardized to the control group and were presented as the mean ± standard deviation of three independent experiments. The blots were cropped and the gels were run under the same experimental conditions. *P < 0.05.

Two computational methods (TargetScan and PicTar) were used to identify miR-34a targets. Among the hundreds of predicted targets, PPARα is closely associated with lipid metabolism in NAFLD^15^,^17^,^21^ and was chosen for further validation in 293T cells using luciferase reporter assays. Figure 3A shows the predicted miR-34a-binding sites in 3′ UTR of *PPARα*.

Furthermore, the wild-type *PPARα*, or a mutant P*PARα*, with the miRNA seed sequence deleted, was co-transfected with miR-34a mimics into 293T cells. The miR-34a mimics suppressed the luciferase activity of the wide-type *PPARα* 3′ UTR 5080 site by 44%. However, full mutation of the *PPARα* 3′ UTR 5080 site seed sequence abrogated the repressive ability of miR-34a, demonstrating the specificity of the miR-34a target sequence in *PPARα* (Fig. 3B).

The ability of miR-34a in regulating the endogenous *PPARα* gene and protein expression was tested. The mRNA and protein levels of PPARα were both increased in L02 cells transfected with the miR-34a inhibitor and cultured for 24 h complete medium and 24h FFA medium, compared with the cells transfected with the NC duplex (Fig. 3C and Figure S1D). In addition, the expression of PPARα was increased in HFD-induced hepatic steatosis mice treated with the miR-34a inhibitor compared to the HFD-induced hepatic steatosis mice treated with the NC duplex (Fig. 3D and Figure S1E). These data suggested that miR-34a downregulated the expression of PPARα by targeting the *PPARα*-3′ UTR 5080 site to facilitate translational repression or mRNA degradation.

### Altered SIRT1 protein expression in L02 cells and liver tissues of mice

The protein level of SIRT1 was increased in L02 cells transfected with the miR-34a inhibitor compared with the cells transfected with the NC duplex (Fig. 3C) and the expression of SIRT1 was increased in HFD-induced hepatic steatosis mice treated with the miR-34a inhibitor compared to the HFD-induced hepatic steatosis mice treated with the NC duplex (Fig. 3D).

### Altered PPARα target metabolic genes expression in L02 cells and liver tissues of mice

**Figure 4:**
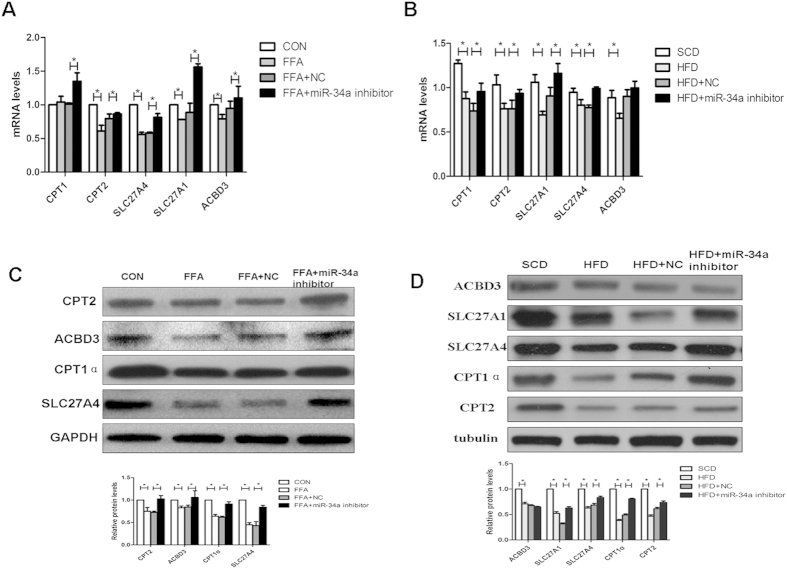
PPARα targets metabolic gene expression in L02 cells and mouse liver tissues. (**A**) QPCR detection of CPT1, CPT2, SLC27A4, SLC27A1 and ACBD3 mRNA in L02 cells or (**B**) liver tissues. Internal control: GAPDH. (**C**) Western blotting analysis of CPT1α, CPT2, SLC27A4 and ACBD3 proteins in L02 cells. GAPDH was detected as a loading control. (**D**) Western blotting analysis of CPT1α, CPT2, SLC27A4, SLC27A1 and ACBD3 proteins in liver tissues. Tubulin was detected as a loading control. The results were standardized to the control group and are the mean ± standard deviation of three independent experiments. The blots were cropped and the gels were run under the same experimental conditions. *P < 0.05.

When the miR-34a inhibitor was transfected or injected, the expression of fatty acid β-oxidation-related genes including CPT1 and CPT2 were significantly increased compared to NC in cells or in liver tissues (Fig. 4A-D). Interestingly, the expression of liver fatty acid transport-related genes, including SLC27A4, ACBD3 and SLC27A1, were significantly reduced in FFA induced steatosis cells or HFD induced steatotic liver compared with control groups (Fig. 4A-D). When transfected or injected with the miR-34a inhibitor, the expression of ACBD3, SLC27A4 and SLC27A1 were significantly increased compared with the NC, but the expression of ACBD3 in C57BL/6 liver tissue did not increased after injecting with the miR-34a inhibitor (Fig. 4A-D), indicating that they could be responsible, or contribute to, the observed phenotype. In summary, the miR-34a inhibitor significantly altered the expressions of genes related to metabolic pathways *in vitro* and *in vivo*.

### Inhibition of miR-34a expression increased the AMPK phosphorylation pathway

**Figure 5:**
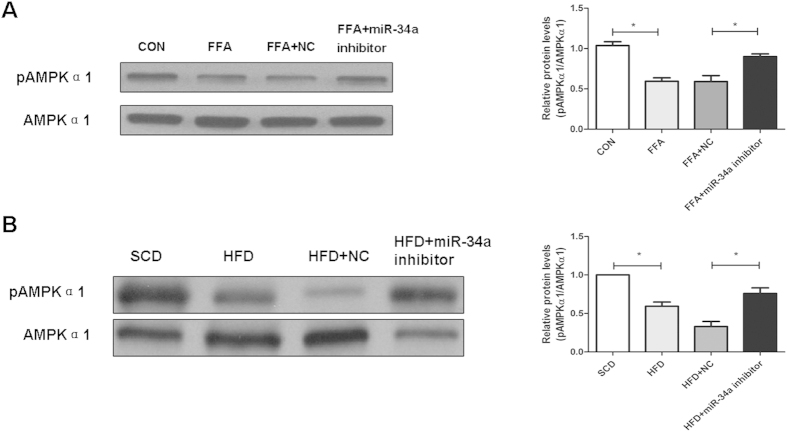
Activated AMPK pathway in L02 cells and mouse liver tissues. Western blotting for phospho-AMPKα1 catalytic subunit in liver extracts from mice treated with miR-34 inhibitor or NC *in vitro* for 24 hours or *in vivo* for 4 weeks. The miR-34a inhibitor activated the AMPK pathway. The results were standardized to the control group and presented as the mean ± standard deviation of three independent experiments. The blots were cropped and the gels were run under the same experimental conditions. *P < 0.05.

To elucidate the mechanism by which the miR-34a inhibitor reduced TG accumulation in L02 cells, we assessed the effect of miR-34a on lipid metabolism pathways. Increased AMPK phosphorylation is associated with enhanced lipid metabolism, especially increased fatty acid β-oxidation^22^,^23^. We found that AMPK phosphorylation was significantly reduced, with the pAMPK/AMPKα1 ratio reduced by about 27% in FFA induced cells compared with control cells (Fig. 5A). The miR-34a inhibitor increased the pAMPK/AMPKα1 ratio compared with the NC. These data suggested a role for AMPK in miR-34a’s effects on fat accumulation *in vitro*.

**Figure 6:**
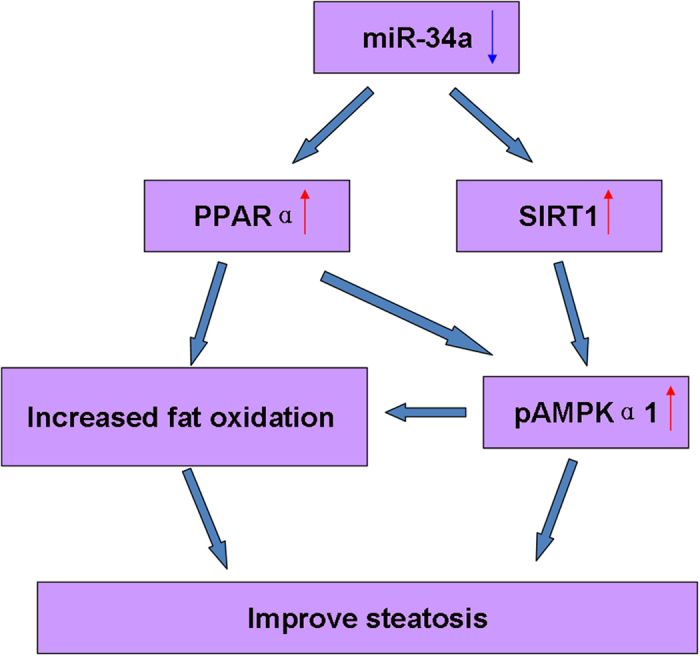
Summarize of miR-34a-PPARα-AMPK regulation in steatosis. First, miR-34a inhibitor treatment increased the expression of PPARα and SIRT1, then PPARα and SIRT1 activated the AMPK pathway. PPARα and pAMPKα1 increased fat oxidation and improve the steatosis finally.

Activated (phosphorylated) AMPK levels were also observed in whole liver extracts of mice treated with the miR-34a inhibitor (2 × 10^7^ TU) compared with the NC. The total AMPKα1 protein level was unchanged in the miR-34a inhibitor-treated mice, but the levels of phosphorylated AMPKα1 were increased nearly 2-fold compared with the NC (Fig. 5B). Figure 6 showed the summarizing of miR-34a-PPARα-AMPK regulation in steatosis.

## Discussion

NAFLD is a multistep process and its pathogenesis has not been clearly delineated. In the present study, we identified a role of miR-34a in regulating the degree of steatosis by directly targeting PPARα. To the best of our knowledge, this has not been done before. We aimed to examine the importance of miR-34a for liver function by inhibiting miR-34a with an antisense oligonucleotide. Additionally, we have uncovered an unexpected role for miR-34a in regulation of hepatic lipid metabolism. This newly identified miR-34a-PPARα pathway provides a novel clue to the pathogenesis of steatosis formation in NAFLD. MiR-34a might become a target for the treatment of NAFLD.

The expression of miR-34a is increased in hepatic fibrosis^24^, HCV infection^10^, alcoholic liver disease^25^, HCC^26^,^27^,^28^ and NAFLD^29^,^30^. However, it was unclear how miR-34a contributes functionally to the development of NAFLD. Our data demonstrated that miR-34a was upregulated in livers of NAFLD mice, and this increased expression was primarily detected in FFA-induced steatosis hepatocytes, suggesting that increased expression of miR-34a in hepatic steatosis is an important part of a protective negative regulatory feedback mechanism aimed at limiting disease progression by preventing excessive lipid accumulation in the liver.

Several studies indicated that serum level of miR-34a was higher in participants with NAFLD^30^. MiR-34a, apoptosis and acetylated p53 increased with disease severity. UDCA inhibited the miR-34a/SIRT1/p53 pathway^31^. Upregulation of miR-34a was found in livers of streptozotocin-induced diabetic mice, demonstrating that miR-34a is strongly dysregulated in NAFLD. The development of NASH was accompanied by prominent changes in the expression of miRNAs, including miR-29c, miR-34a, miR-155, and miR-200b. Interestingly, changes in the expression of these miRNAs and the protein levels of their targets, including Cebp-β, Socs 1, Zeb-1, and E-cadherin, in the livers of DBA/2J mice fed MCD were more pronounced compared with control mice. These results showed that alterations in the expression of miRNAs are prominent events during development of NASH, and strongly suggest that the severity of and susceptibility to NASH may be determined by variations in miRNA expression response^12^.

MiRNAs negatively regulate their target mRNAs posttranscriptionally by binding to the 3′ UTR. In the present study, TargetScan and PicTar were used to identify potential targets of miR-34a. We then focused on PPARα because it plays a pivotal role in hepatic steatosis and metabolic pathways^17^,^21^. We confirmed the regulation of the predicted miR-34a targeted mRNAs by measuring both mRNA and protein levels in cells and mice after miR-34a inhibitor treatment, indicating that miRNA regulation could result in mRNA degradation and translational regulation of the predicted targets. Furthermore, the specific binding effect between miR-34a and the wild-type luciferase construct verified the potential post-transcriptional regulation of miR-34a on PPARα.

SIRT1, a mammalian ortholog of Sir2 (silent information regulator 2), is an NAD-dependent deacetylase that acts as a master metabolic sensor of NAD and modulates cellular metabolism^32^,^33^. In our research, we also found that protein levels of SIRT1 were significantly increased in cells and mice liver tissues after miR-34a inhibitor treatment. These results were consistent with the previous research. Choi *et al.*^34^, recently showed that hepatic miR-34a, which is elevated in obesity, directly targets and decreases SIRT1 expression.

Given the abundance of miR-34a in the liver, it was expected to play an important role in maintenance of liver lipid metabolism. In the present study, H&E staining and Oil red O staining both demonstrated that the miR-34a inhibitor attenuated the steatosis in the liver tissues and in L02 cells. In accordance with morphological findings, TG levels in L02 cells and liver tissues were reduced in the miR-34a inhibitor group. Furthermore, liver weight/body weight ratio (liver index) was also reduced significantly in the miR-34a inhibitor group. In HFD group, the liver index was reduced significantly compared to SCD group, which may be due to the body weight gain was much greater than the liver weight gain.

Surprisingly, the most apparent consequence of miR-34a inhibition in HFD mice over 4 weeks was a decrease in plasma AST, by about 15% in mice compared to NC after 4 weeks of HFD treatment. These results will be helpful for studies in evaluating the prospects for therapeutically inhibiting miR-34a to lower AST levels in humans.

The mitochondrial carnitine system plays a crucial role in β-oxidation. CPT1 catalyzes the esterification of long-chain acyl-CoAs to L-carnitine for transport into mitochondria for FAO. The acyl-carnitine conjugate is converted back to the acyl-CoA ester inside mitochondria by CPT2^35^. In the present study, we found that the miR-34a inhibitor activates AMPK, an enzyme that functions as a fuel gauge to monitor the status of cellular energy, and activates the expression of genes of FAO. The activated AMPK increased CPT1 and CPT2 levels, leading to an increase in the transport of FA into mitochondria and stimulating the oxidation of mitochondrial palmitoyl-CoA. These observations begin to explain the mechanisms for increased FAO after inhibition of miR-34a, identifying AMPK as a mediator of increased FAO in liver of miR-34a inhibitor treatment.

In cells, there are several families of lipid-binding proteins, such as Acyl-coenzyme A binding domains (ACBDs), fatty acid transport proteins (FATPs or SLC27As) and fatty acid binding proteins (FABPs), which function coordinately to regulate homeostasis and the function of fatty acids. In our study, the expression of energy metabolism-related genes including SLC27A1, SLC27A4 and ACBD3, which act downstream of PPARα, were increased in the L02 cells and the liver of miR-34a inhibitor treated mice, which suggested some of the differences in fat accumulation may have been caused by increased FA uptake into the cells^36^.

The central metabolic sensor AMPK is a key regulatory enzyme that promotes ATP-generating pathways, such as FAO, and inhibits energy-storage pathways, such as FA synthesis^22^,^37^. We observed that miR-34a inhibition in cells and mice were consistent with increased AMPK activity. These results support the role of AMPK as a mediator of miR-34a’s effects on PPARα and its target genes. Hou *et al.*^38^ reported that the overexpression of SIRT1 stimulated the basal AMPK signaling in HepG2 cells and in the mouse liver. In our study, miR-34a inhibition also stimulated AMPK signaling though PPARα in cells and mice liver. Therefore, we think miR-34a-mediated PPARα regulation and SIRT1-AMPK pathway may be two separate regulatory mechanisms in steatosis, but there was a certain link between them, beacause both of them stimulated AMPK signaling.

Increased FAO after miR-34a inhibition led to a significant improvement in liver steatosis. These NAFLD cells and mice represent a state of prolonged caloric excess, which results in obesity, hyperlipidemia and hepatic steatosis. Our data suggested that miR-34a played a significant role in promoting lipid synthesis and inhibiting FAO in liver, and as such, may be a therapeutic target for metabolic diseases.

In summary, our study demonstrated that miR-34a was upregulated in NAFLD and has a novel regulatory role as an important regulator of lipid metabolism. PPARα, a direct target of miR-34a, was upregulated after miR-34a inhibitor treatment, which induced transcriptions of CPT1 and CPT2 (both involved in fatty acid β-oxidation), SLC27A1, SLC27A4 and ACBD3 (all involved in fatty acid transport), causing the decrease of TG, liver index, as well as the activated AMPK pathway. Furthermore, SIRT1 was also upregulated after miR-34a inhibitor treatment. The present study contributes to our understanding the pathogenesis of steatosis formation in NAFLD and may lead to novel therapies for NAFLD that target upstream molecules at the miRNA level.

## Additional Information

**How to cite this article**: Ding, J. *et al.* Effect of miR-34a in regulating steatosis by targeting PPARα expression in nonalcoholic fatty liver disease. *Sci. Rep.* **5**, 13729; doi: 10.1038/srep13729 (2015).

## Supplementary Material

Supplementary Information
