# Supplementary material for: Effect of miR-34a in regulating steatosis by targeting PPARα expression in nonalcoholic fatty liver disease
Source: Sci Rep. 2015 Sep 2;5:13729. doi: 10.1038/srep13729 (PMC4557122; doi:10.1038/srep13729)
Supplement: Supplementary Information [file srep13729-s1.pdf]

Effect of miR-34a in regulating steatosis by targeting PPARα expression in nonalcoholic fatty liver disease

Jiexia Ding[#], Meng Li[#], Xingyong Wan, Xi Jin, Shaohua Chen, Chaohui Yu, Youming Li[*]

Supplementary Materials and Methods

Hematoxylin and eosin and Oil red O staining

The mice were killed after an overnight fast. Livers were fixed in 10% formalin for 24 hours or kept freshly frozen. Formalin-fixed paraffin-embedded liver sections (5μm) were stained with hematoxylin and eosin (HE). Oil red O staining was carried out on frozen liver sections (10μm). Cells or liver tissue were fixed with 10% formaldehyde for 10 minutes. After two washes in PBS, cells were stained for 10 minutes in freshly diluted Oil red O solution. The dishes were then rinsed in water and counterstained with hematoxylin for 5 minutes. Representative photomicrographs were captured at 200× or 400× magnification using a system incorporated in the microscope.

Triglyceride assay

Intracellular and liver triglycerides were assayed using a triglyceride assay kit (GPO-POD; Applygen Technologies Inc., Beijing, China) according to the manufacturer's recommended protocol.

RNA extraction and real-time quantitative PCR (qPCR)

MiRNA was extracted from mouse liver tissues or L02 cell lines using RNAiso for small RNA kit (Takara, Dalian, China). Before detection, miRNA was polyadenylated and reverse transcribed into cDNA using a One Step PrimeScript ® miRNA Cdna

Synthesis Kit (Takara, Dalian, China). Total RNA was extracted using TRIzol reagent (Takara, Dalian, China) and reverse transcribed into cDNA using a PrimeScript® RT reagent Kit (Takara, Dalian, China). The resulting cDNA was detected with an ABI 7500 real-time PCR System (Applied Biosystems, Carlsbad, USA) with SYBR Green (Takara, Dalian, China). The small nuclear RNA U6 and GAPDH mRNA were used as internal controls for miRNA and mRNA detection, respectively. The primers for q-PCR were obtained from Sango Biotech (Shanghai, China) and the sequences of human L02 cell lines were as follows: U6 (forward: 5'-TGC GGG TGC TCG CTT CGG CAG C-3'), PPARα (forward: 5'-CGG TGA CTT ATC CTG TGG TCC-3', reverse: 5'-CCG CAG ATT CTA CAT TCG ATG TT-3'), SLC27A4 (forward: 5'-GGA CCC AGG TGG GAT TCTC-3', reverse: 5'-CGC GCC TGA TGG TCT TGA T-3'), SLC27A1 (forward: 5'-CTC AGG TGA CGT GCT AGT GAT-3', reverse: 5'-GCT CCT GGT ATA TCG CGT TGG-3'), ACBD3 (forward: 5'-ACA GTA TCC AGG GAA CTA CGA A-3', reverse: 5'-GTT TCT GTA ATG CTG CCT GTT G-3'), CPT1 (forward: 5'-TCC AGT TGG CTT ATC GTG GTG-3', reverse: 5'-TCC AGA GTC CGA TTG ATT TTT GC-3'), CPT2 (forward: 5'-CAT ACA AGC TAC ATT TCG GGA CC-3', reverse: 5'-AGC CCG GAG TGT CTT CAG AA-3') and GAPDH (forward: 5'-TCA ACG ACC ACT TTG TCA AGC TCA-3', reverse: 5'-GCT GGT GGT CCA GGG GTC TTA CT-3'), and the sequences of mouse were as follows: U6 (forward: 5'-TGC GGG TGC TCG CTT CGG CAG C-3'), PPARα (forward: 5'-AGA GCC CCA TCT GTC CTC TC-3', reverse: 5'- ACT GGT AGT CTG CAA AAC CAA A-3'), SLC27A4 (forward: 5'-ACT GTT CTC CAA GCT AGT GCT-3', reverse:

5'-GAT GAA GAC CCG GAT GAA ACG-3'), SLC27A1 (forward: 5'-CGC TTT CTG CGT ATC GTC TG-3', reverse: 5'-GAT GCA CGG GAT CGT GTC T-3'), ACBD3 (forward: 5'-GAG GAG CTT TAC GGC CTG G-3', reverse: 5'-CTT ATG CAG TGC CAC GAA CTT-3'), CPT1 (forward: 5'-CTC CGC CTG AGC CAT GAA G-3', reverse: 5'-CAC CAG TGA TGA TGC CAT TCT-3'), CPT2 (forward: 5'-CAG CAC AGC ATC GTA CCC A-3', reverse: 5'-TCC CAA TGC CGT TCT CAA AAT-3') and GAPDH (forward: 5'-AGG TCG GTG TGA ACG GAT TTG-3', reverse: 5'-GGG GTC GTT GAT GGC AAC A-3'). The primer sequence of miR-34a for qPCR was obtained from Takara (Dalian, China). Data were presented as RQ values ($2^{-\triangle\triangle CT}$) with expression relative to endogenous control sample.

MiR-34a target prediction and validation

MiR-34a target prediction was carried out using the algorithms TargetScanHuman V6.2 (http://www.targetscan.org/vert_61/) and PicTar (http://pictar.mdc-berlin.de). In order to validate the candidate miR-34a predicted targets, luciferase reporter assays were carried out. The 3' UTRs of PPARα containing miR-34a binding sites(wild) or mutation seed sites (mut) were cloned downstream of the luciferase reporter in the pmirGLO Dual-Luciferase miRNA Target Expression Vector (Promega, Madison, USA). The 293T cells were plated in 24-well plates and co-transfected with 50 nM miRNA mimic or dsControl RNA and with 100 ng of the luciferase vector (pmirGLO). The cells were harvested 48h post-transfection, and the luciferase activity was measured using the Dual-Glo luciferase assay kit (Promega). The transfection of hsa-miR-34a mimics (sense 5'-UGG CAG UGU CUU AGC UGG UUG U-3';

antisense 5'-AAU CAG CUA AGA CAC UGC CAU U-3', chemically synthesized by Invitrogen, Carlsbad, USA) was performed using Lipofectamine 2000 (Invitrogen, Carlsbad, USA) according to the manufacturer's protocol. The nonsense double-strand RNA (dsRNA; sense 5'-UUC UCC GAA CGU GUC ACG UTT-3'; antisense 5'-ACG UGA CAC GUU CGG AGA ATT -3', chemically synthesized by Invitrogen, Carlsbad, USA).

Western blotting analyses

Briefly, following the various treatments, the cells and the liver tissue were washed and lysed with lysis buffer. The protein concentration in the resulting lysates was determined using the bicinchoninic acid protein assay kit (Applygen Technologies Inc., Beijing, China). Approximately 30-50μg of protein was denatured and resolved by electrophoresis in 10-15% Tris–glycine polyacrylamide gels and transferred to polyvinylidene fluoride (PVDF) membranes (Massachusetts, USA). The membranes were blocked with 5% nonfat milk and incubated overnight with antibodies against PPARα, SIRT1, tubulin, SLC27A4, SLC27A1, ACBD3, CPT1α, CPT2 (all from abcam, UK), phosphorylated AMPKα1(from Cell Signaling Technology, Beverly, USA) GAPDH and AMPKα1 (both from Epitomics, Burlingame, USA) at dilutions specified by the manufacturer. The membranes were washed three times in TBST and incubated with the corresponding horseradish peroxidase (HRP)-conjugated secondary antibody at a 1:5000 dilution for 1h. After washing three times with TBST, the bound secondary antibody was detected using a potent chemiluminescence (ECL) kit (Multisciences, Hangzhou, China) and quantitated using Quantity One software.

Statistical Analysis

Results are expressed as means $\pm$ standard deviation. The significance of the difference in means was determined by two-tailed Student *t* test and ANOVA test. Values of $P < 0.05$ were considered significant and are indicated by asterisks in the figures.

Supplementary Figure Legends

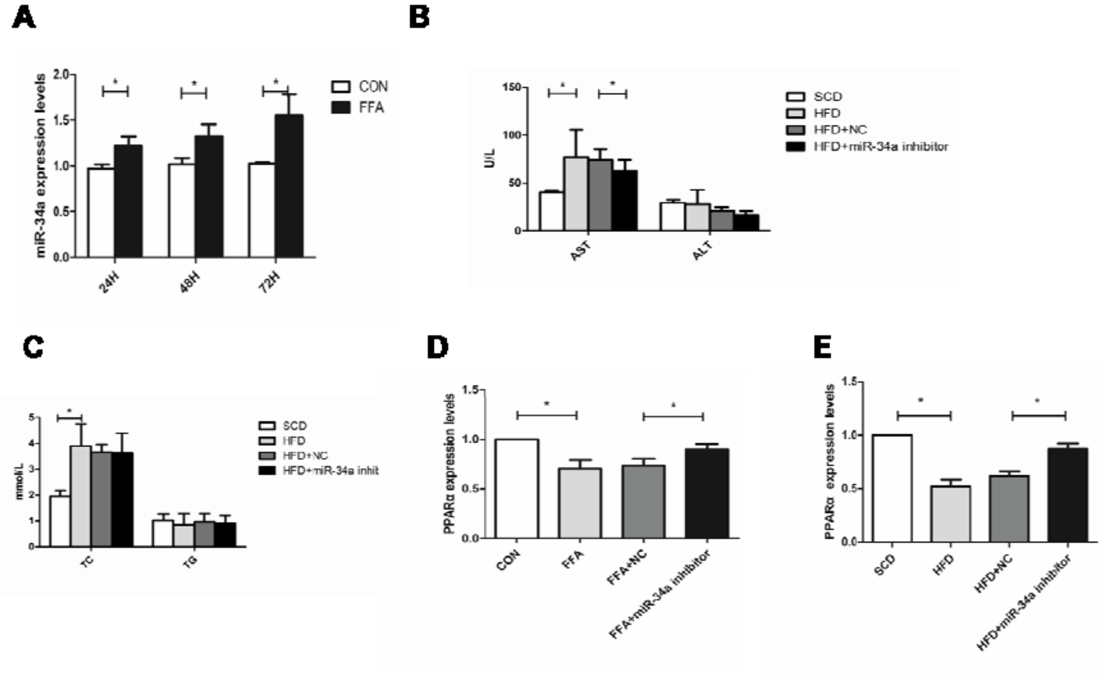

Figure S1. The expression of miR-34a in L02 cells of NAFLD models, influence of miR-34a inhibitor on plasma biochemistry induced by HFD and gene expression of PPARα following inhibition of miR-34a in L02 cells and liver tissues. (A) The expression of miR-34a in L02 cells treated with FFA and normal cultures. (B and C) Influence of miR-34a inhibitor on plasma biochemistry induced by HFD. (D and F) Decreased gene expression of PPARα was observed in steatosis L02 cells, and increased in the miR-34a inhibitor treated group in L02 cells and liver tissues. The PPARα mRNA was detected by qPCR; GAPDH was used as an internal control. Representative results from three independent experiments are shown. Data are mean ± standard deviation in A, B, C, D and E. **P* < 0.05.
